# Newly identified colistin resistance genes, *mcr-4* and *mcr-5*, from upper and lower alimentary tract of pigs and poultry in China

**DOI:** 10.1371/journal.pone.0193957

**Published:** 2018-03-14

**Authors:** Li Chen, Jilei Zhang, Jiawei Wang, Patrick Butaye, Patrick Kelly, Min Li, Feng Yang, Jiansen Gong, Afrah Kamal Yassin, Weina Guo, Jing Li, Chunlian Song, Chengming Wang

**Affiliations:** 1 College of Veterinary Medicine, Yangzhou University, Yangzhou, Jiangsu, China; 2 Department of Biosciences, Ross University School of Veterinary Medicine, Basseterre, St. Kitts, West Indies; 3 Department of Pathology, Bacteriology and Poultry diseases, Faculty of Veterinary Medicine, Ghent University, Ghent, Belgium; 4 Poultry Institute, Chinese Academy of Agricultural Sciences, Yangzhou, Jiangsu, China; 5 Department of Food Hygiene and safety, Faculty of Public and Environmental Health, University of Khartoum, Khartoum, Sudan; 6 College of Animal Science, Anhui Science and Technology University, Bengbu, Anhui, China; 7 College of Animal Science & Technology, Yunnan Agricultural University, Kunming, Yunnan, China; 8 Department of Pathobiology, College of Veterinary Medicine, Auburn University, Auburn, AL, United States of America; Kansas State University, UNITED STATES

## Abstract

Antimicrobial resistance against colistin has emerged worldwide threatening the efficacy of one of the last-resort antimicrobials used for the treatment of *Enterobacteriacea*e. To investigate the presence of the recently identified colistin resistance genes (*mcr-4*, *mcr-5*) in China, we established PCRs to detect *mcr-4* and *mcr-*5 on 213 anal and 1,339 nasal swabs from apparently healthy pigs (n = 1,454) in nine provinces, and 1,696 cloacal and 1,647 oropharyngeal samples from poultry (n = 1,836) at live-bird markets in 24 provinces of China. The prevalence of the *mcr-4* in swine swabs (41.4%; 642/1,552) was significantly higher than in swabs from poultry (11.5%; 384/3,343). The *mcr-4* gene was found in geese (49.5%, 54/109), chickens (17.2%, 257/1,498), pigeons (17.2%, 17/99) and ducks (15.4%, 20/130). In a similar trend, the prevalence of the *mcr-5* in swine swabs (33.1%; 514/1552) was significantly higher than in swabs from poultry (5.6%; 187/3,343). The *mcr-5* was identified in geese (17.4%, 19/109), chickens (9.9%, 148/1,498), ducks (7.7%, 10/130) and pigeons (3%, 3/99). The *mcr-*4 prevalence in the nasal swabs from pigs (59.2%, 58/98) was significantly higher than that in anal swabs (29.6%, 29/98) (P<0.001). Similarly, the *mcr-5* prevalence in the nasal swabs from pigs (61.2%, 60/98) was significantly higher than in anal swabs (44.9%, 44/98) (P = 0.02), and significantly higher in oropharyngeal swabs (7.2%, 109/1,507) than in the cloacal swabs (3.7%, 56/1,507) (P<0.001). This study further confirms the presence of the *mcr-4* and *mcr-5* in animals and indicates these genes are prevalent and widespread in food producing animals (pig and poultry) in China. Future studies are needed to characterize the bacteria carrying the *mcr-4* and *mcr-5* and their locations on plasmids and/or the bacterial chromosomes, and determine co-resistances in the *mcr-4* and *mcr-5* positive strains.

## Introduction

Antimicrobial resistance is one of the most serious global threats to human health, especially the multiple drug resistant-pathogens of ESKAPE group (*Enterococcus faecium*, *Staphylococcus aureus*, *Klebsiella pneumoniae*, *Acinetobacter baumannii*, *Pseudomonas aeruginosa* and *Enterobacter* spp.) [1]. The reintroduction of the older and less user-friendly antibiotics such as colistin is an option for treatment of the infections with Gram-negative ESKAPE bacteria in humans [2]. However, the efficiency of colistin treatment is compromised by the presence of an increasing number of mobile colistin resistance (*mcr*) genes. After *mcr-*1 was first described in 2016, *mcr-2* and *mcr-3* genes have been found to occur very widely [3–22]. Up to the current writing of this manuscript, another two *mcr* genes (*mcr-4* and *mcr-5*) were identified in *Salmonella* [23–26], but little is known about the prevalence of these two genes. In this study, PCRs were established to the presence of *mcr-4* and *mcr-5* genes in swine and poultry swab samples in China.

## Materials and methods

### Swab samples from swine and poultry

This study was reviewed and approved by the Institutional Animal Care and Use Committee of Yangzhou University College of Veterinary Medicine (YZU-CVM IACUC 2013#87, YZU-CVM IACUC 2015#57).

Nasal (n = 1,339) and anal (n = 213) swabs collected from apparently healthy pigs (n = 1,454) from nine provinces of China in 2016 (Table 1, S1 Table) [27] were used in this study to investigate the prevalence of *mcr-4* and *mcr-5*. In addition, oropharyngeal and cloacal samples were obtained from poultry (n = 1,836) at 38 live-bird markets in 24 provinces in China between 2014 and 2015 (Table 1) [28], and 1,647 oropharyngeal and 1,696 cloacal samples from 1,836 birds were used in this investigation. The swabs from pigs and poultry were collected into tubes containing 400μl DNA/RNA Stabilization Buffer (Roche Molecular Biochemicals, IN, USA), and frozen at -80°C until DNA extraction. Swabs were centrifuged in the DNA/RNA Stabilization Buffer (3,000×g, 4°C for 5 min), and DNAs were extracted from the supernatants using the High Pure PCR Template Preparation Kit (Roche Diagnostic, USA) as described before [22, 28]. All samples from previous studies that had sufficient residual DNA extract were included in this investigation.

**Table 1:** Prevalences of *mcr-4* and *mcr-5* in swabs from pigs and poultry.

Province	pig		chicken		duck		goose		pigeon	
	*mcr-4*	*mcr-5*	*mcr-4*	*mcr-5*	*mcr-4*	*mcr-5*	*mcr-4*	*mcr-5*	*mcr-4*	*mcr-5*
Anhui			4/34	0/34						
Fujian			6/35	1/35	2/33	1/33			0/13	0/13
Gansu			2/57	0/57						
Guangdong	8/40	11/40	24/65	10/65	0/4	0/4				
Guangxi			23/130	12/130	3/10	1/10				
Hainan			6/70	15/70						
Hebei			26/96	3/96	2/6	0/6			10/34	1/34
Heilongjiang	15/60	4/60								
Henan	35/63	8/63	11/56	12/56	1/7	0/7	3/7	1/7		
Hubei			13/64	3/64	4/6	0/6				
Hunan			0/70	8/70						
Inner Mongolia			9/65	6/65			0/5	0/5		
Jiangsu	238/590	109/590	11/154	20/154	3/31	5/31	5/9	7/9	5/46	1/46
Jiangxi			18/49	24/49	1/11	3/11	1/9	2/9		
Jilin	33/63	2/63	24/70	8/70						
Liaoning			10/37	3/37	0/7	0/7			2/6	1/6
Shaanxi			0/70	0/70						
Shandong	3/60	8/60	1/59	0/59	1/3	0/3	1/8	0/8		
Shanghai	51/53	6/53					44/70	9/70		
Shanxi			20/20	4/20						
Sichuan			9/70	3/70						
Tibet			5/30	0/30						
Xinjiang			17/70	4/70						
Yunnan	41/130	27/130	10/70	7/70						
Zhejiang	197/395	303/395	8/57	5/57	3/12	0/12	0/1	0/1		
Total	621/1454	478/1454	257/1498	148/1498	20/130	10/130	54/109	19/109	17/99	3/99
	42.7%	32.9%	17.2%	9.9%	15.4%	7.7%	49.5%	17.4%	17.2%	3.0%

### Real Time PCRs for *mcr-4* and *mcr-5*

The representing nucleotide sequences for *mcr-4* (MF543359) and *mcr-5* (KY807920, KY807921) were obtained from the NCBI, and were aligned using the Clustal Multiple Alignment Algorithm. Based on the alignment, two *mcr-4* PCRs were designed, one with a short 206-bp amplicon (forward: 5’-AGGTTTAGTGTTCGGGTTACGACTGG-3’; reverse: 5’-GCATTGGGATAGTCGCCTTTTTTTACTA-3’) and another with a long 1,165-bp amplicon (forward: 5’-AATTGTCGTGGGAAAAGCCGC-3’; reverse: 5’-CTGCTGACTGGGCTATTACCGTCAT-3’). The short amplicon PCR was used to establish prevalence data and positive samples were then tested with the long amplicon qPCR for phylogenetic studies.

Similarly, two *mcr-5* PCRs were designed, the one producing a short 271-bp amplicon (forward: 5’-GTGAAACAGGTGATCGTGACTTACCG-3’, reverse: 5’-CGTGCTTTACACCGATCATGTGCT-3’) and the other a long 1,251-bp amplicon (forward: 5’-ACTCGACTGCCACCAGATCATCG-3’, reverse: 5’-CGCTGGAGTGTCAAGCCACTACTG-3’). The short amplicon PCR was used to establish prevalence data and positive samples were then tested with the long amplicon PCR for phylogenetic studies.

The specificity of the primers for the *mcr-4* and *mcr-5* PCRs was verified by BLASTN and DNA sequencing of the amplicons obtained using synthesized plasmids containing portions of the target *mcr-4* and *mcr-5* that were cloned into the *SacI* site (Takara Biothechnology, Dalian, China). The sensitivity of the *mcr-4* PCRs and *mcr-5* PCRs was determined by amplifying dilutions of the synthesized plasmids. The PCRs were quantified using the PicoGreen DNA fluorescence assay (Molecular Probes, Eugene, OR, USA) with standards prepared with the synthesized plasmids (10^4^, 10^3^, 10^2^, 10^1^, and 10^0^ copies/reaction) (Genscript, Nanjing, China).

The PCRs were performed on a Roche LightCycler 480 II PCR instrument. The PCRs with short amplicons were SYBR based, and were used to determine the presence of *mcr-4* and *mcr-5* in swabs in this study. The positive samples determined by PCRs were further amplified by PCRs with long amplicons. The PCR products of both short and long amplicons were sequenced using forward and reverse primers (BGI, Shanghai, China).

### Phylogenetic analysis

The *mcr* sequences obtained from this study and those from GenBank for the *mcr-4* and *mcr-5* were aligned used the MEGA 6.0 software to compare their similarities.

### Statistical analysis

Multiple Pearson Chi-square test was used to compare differences between animal species as well as between oropharyngeal/anal and oral/nasal swabs with Bonferroni adjusted p-values. P value below 0.05 was considered significantly different.

## Results

### Establishment of PCRs for *mcr-4* and *mcr-5*

The SYBR-based real-time PCRs detected the positive control plasmids containing the target *mcr-4* and *mcr-5* sequences with a detection limit of one gene copy per reaction. The detection limit was 10 copies per reaction for the *mcr-4* and *mcr-5* PCRs with long amplicons. The specificity of the PCRs was verified by gel electrophoresis and DNA sequencing.

### Prevalence of *mcr-4*

Overall, the prevalence of the *mcr-4* as detected by the short amplicon Real Time PCR in swine swabs (41.4%; 642/1,552) was significantly higher than in swabs from poultry (11.5%; 384/3,343) (P<0.01). The *mcr-4* positivity determined by PCR was 17.4% for the anal (37/213) and 45.2% of the nasal swabs (605/1,339) in pigs, and 11.5% for the cloacal (195/1,696) and 11.5% of the oropharyngeal swabs (189/1,647) in poultry (Table 2, S2–S6 Tables).

**Table 2:** Comparison of the prevalence of *mcr-4* and *mcr-5* in nasal/oropharyngeal and anal/cloacal swabs of pigs and poultry.

Positivity of *mcr*	pig			chicken			duck			goose			pigeon		
	nasal	anal	total*	Oro**	cloacal	total	oro	cloacal	total	oro	cloacal	total	oro	cloacal	total
	(1339)	(213)	(1454)	(1350)	(1383)	(1498)	(122)	(122)	(130)	(109)	(109)	(109)	(66)	(82)	(99)
*mcr-4*	605	37	621	137	143	257	9	11	20	37	30	54	6	11	17
	45.2%	17.4%	42.7%	10.1%	10.3%	17.2%	7.4%	9.0%	15.4%	33.9%	27.5%	49.5%	9.1%	13.4%	17.2%
*mcr-5*	455	59	478	93	58	148	8	3	10	16	6	19	1	2	3
	34.0%	27.7%	32.9%	6.9%	4.2%	9.9%	6.6%	2.5%	7.7%	14.7%	5.5%	17.4%	1.5%	2.4%	3.0%
*mcr-4* and *mcr-5*	255	27	266	28	6	33	3	1	4	9	2	10	1	0	1
	19.0%	12.7%	18.3%	2.1%	0.4%	2.2%	2.5%	0.8%	3.1%	8.3%	1.8%	9.2%	1.2%	0.0%	1.0%

* Total means the total number of the assayed animals. Under the column of Total, when one of the Nasal/Oral and Anal/cloacal swabs was positive, this animal was considered to be positive

** oro indicates oropharyngeal swab

Overall, poultry in 21 of the 24 provinces of China we studied were positive for the *mcr-4*. The *mcr-4* gene was found in swabs all four of the poultry species we studied, geese (49.5%, 54/109), chickens (17.2%, 257/1,498), pigeons (17.2%, 17/99), and ducks (15.4%, 20/130). The prevalence of the *mcr-4* in cloacal swabs from geese (27.5%, 30/109) was significantly higher than that from pigeons (13.4%, 11/82), chickens (10.3%, 143/1,383), and ducks (9%, 11/122). Similarly, the oropharyngeal swabs from geese (33.9%, 37/109) were most commonly positive by *mcr-4* PCR than those from chickens (10.1%; 137/1,350), pigeons (9.1%; 6/66) and ducks (7.4%; 9/122) (Table 1, S3–S6 Tables).

### Prevalence of *mcr-5*

Overall, the *mcr-5* short amplicon PCR were significantly more commonly positive with swabs from pigs (nasal swabs: 34.0%, 455/1,339; anal swabs: 27.7%, 59/213) than with swabs from poultry (oropharyngeal swabs: 7.2%, 118/1,647; cloacal swabs: 4.1%, 69/1,696) (Table 1, Table 2, S2–S6 Tables).

Positive *mcr-5* PCR were obtained from poultry sampled in 19 of the 24 provinces with all four species being positive, mainly geese (17.4%, 19/109), chickens (9.9%, 148/1,498), ducks (7.7%, 10/130), and pigeons (3%, 3/99). The oropharyngeal swabs from geese were most commonly *mcr-5* positive (14.7%, 16/109) followed by those from chickens (6.9%, 93/1,350), ducks (6.6%, 8/122), and pigeons (1.5%, 1/66). The prevalence of the *mcr-5* in cloacal swabs was 5.5% in geese (6/109), 4.2% in chickens (58/1,383), 2.5% in ducks (3/122), and 2.4% in pigeons (2/82) (Table 1, S3–S6 Tables).

### Co-occurrence of *mcr-4* and *mcr-5*

We identified both the *mcr-4* and *mcr-5* in swabs from 48 poultry (2.6%, 48/1,836), including 33 chickens (oropharyngeal: 28; cloacal: 6), four ducks (oropharyngeal: 3; cloacal: 1), ten geese (oropharyngeal: 9; cloacal: 2) and one pigeon (oropharyngeal: 1; cloacal: 0) (Table 2). Both the *mcr-4* and *mcr-5* were detected in 18.3% (266/1,454) of the pigs with 27 anal swabs (27/213) and 255 nasal swabs (255/1,339) found positive for both genes (Table 2).

### Comparison of the presence of *mcr* in swabs from the upper and lower alimentary tract

**Fig 1:**
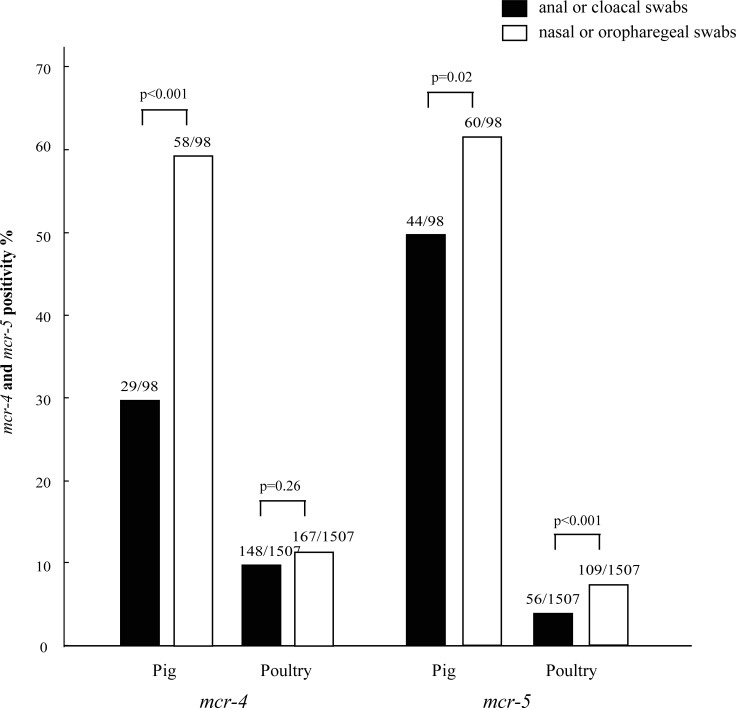
Prevalence of *mcr-4* and *mcr-5* in the upper and lower alimentary system of pigs and poultry. In swabs collected from both locations in 98 pigs and 1,507 poultry, the prevalences of the *mcr-4* and *mcr-5* in pigs and the *mcr-5* in poultry were significantly higher in nasal/oropharyngeal swabs than in the anal/cloacal swabs.

Swabs from both the upper (represented by nasal and oropharyngeal swabs) and lower alimentary tract (represented by anal and cloacal swabs) were available for each of 98 pigs and of 1,507 poultry we studied. Both of the swabs were positive for *mcr-4* in 21 (21.4%, 21/98) of the pigs but only the anal swab was *mcr-4* positive in eight pigs (8.2%, 8/98), and only the nasal swab was *mcr-4* positive in 37 pigs (37.8%, 37/98) (Fig 1, S3 Table). The prevalence of *mcr-4* in the nasal swabs from pigs (59.2%, 58/98) was significantly higher (p<0.001) than that in anal swabs (29.6%, 29/98) (Fig 1).

Both the oropharyngeal and cloacal swabs were *mcr-4* positive in 36 poultry (2.4%: 23 chickens, 13 geese). The cloacal swab was the only positive swab for 112 birds (7.4%: 82 chickens, 10 ducks; 17 geese, 3 pigeons) and the oropharyngeal swab was the only positive swab for 131 birds (8.7%: 98 chickens, 5 ducks, 24 geese, 4 pigeons) (Fig 1, S3–S6 Tables). The prevalence of the *mcr-4* in the oropharyngeal swabs (11.1%, 167/1,507) of poultry was not significantly different from that in the cloacal swabs (9.8%, 148/1,507) (Fig 1).

With respect to the *mcr-5* PCR, 36 of the 98 (36.7%) pigs, from which both nasal and anal swabs were taken, were positive for both swabs; only the anal swab was positive for 8 pigs (8.2%, 8/98) and only the nasal swab positive for 24 pigs (24.5%, 24/98) (Fig 1, S2 Table). The prevalence of the *mcr-5* in the nasal swabs from these pigs (61.2%, 60/98) was significantly higher (p = 0.02) than in anal swabs (44.9%, 44/98) (Fig 1).

Of the 1,507 poultry from which both oropharyngeal and cloacal swabs were collected, 7 birds (0.5%: 3 chickens, 1 duck, 3 geese) were *mcr-5* positive in both swabs. Forty-nine birds (3.3%: 43 chickens; 2 ducks, 3 geese, 1 pigeons) were positive to *mcr-5* only for cloacal swabs, and 102 birds (6.8%: 82 chickens, 6 ducks, 13 geese, 1 pigeon) were positive to *mcr-5* in only oropharyngeal swabs (Fig 1, S3–S6 Tables). The prevalence of the *mcr-5* in oropharyngeal swabs (7.2%, 109/1,507) was significantly higher (p<0.001) than in the cloacal swabs (3.7%, 56/1,507) (Fig 1).

### Phylogenetic comparison

We successfully sequenced the products of 26 of the *mcr-4* long amplicon PCRs (9 pigs, 6 chickens, 10 geese, 1 pigeon) and 24 of the *mcr-5* long amplicon PCRs (15 pigs, 6 chickens, 3 geese).

The nucleotide sequences of the *mcr-4* (1,165-bp) we obtained were all identical to one another, and to a sequence obtained from a vaginal swab from a woman in China (MG520404). Further, they had 99.8% similarity (two mismatches) with the first *mcr-4* sequence, reported from a *Salmonella* strain in Italy in 2013 (MF543359). The similarity at the amino acid level (388-aa) with the *Salmonella* pMCR_R3445 strain from Italy was 99.5% (two mismatches).

The nucleotide sequences (1,251-bp) of the *mcr-5* identified in our study were identical to one another and to the first *mcr-5*, sequenced from *Salmonella enterica subsp*. pSE12-02541 (KY807920), as well as the *mcr-5* of *Salmonella enterica subsp*. pSE13-SA01718 (KY807921), and the *mcr-5* in the vaginal swab from a woman in China (MG520405).

All nucleotide sequences were submitted to GenBank with accession numbers MG586909 to MG586912 for *mcr-4* and MG586913 to MG586915 for *mcr-5*.

## Discussion

The usefulness of colistin, the last-resort antibiotic used to treat multidrug resistant Gram-negative bacterial infections, is being compromised with the recent identification of the mobile colistin resistance gene, *mcr-1* [3], and the subsequent finding of *mcr-2*, *mcr-3*, *mcr-4* and *mcr-5* [4–5, 23–26]. The *mcr-1*, *mcr-2* and *mcr-3* have been detected in bacteria or swabs from a variety of hosts in China and elsewhere in the world [3–22] However, there are yet only few reports on *mcr-4* and *mcr-5* [23–26]. Our study shows the *mcr-4* and *mcr-5* occur widely in pigs and poultry in China (Table 1, S2–S6 Tables). The prevalences of the genes we detected using PCR of swabs from animals were considerably higher than those obtained with studies that relied on bacterial isolates [23, 24]. The sensitive and specific PCR we used to detect the mcr-4 and *mcr-5* directly in swabs avoided the limitations introduced by bacterial isolation and the associated underestimation of the prevalence of the *mcr’*s, the so-called ‘phantom resistome’ [29]. Although bacterial isolation for resistance testing is expensive, laborious, time consuming, and limits the resistant strains detected in a sample, it is an important adjunct to detection by molecular methods and enables a more complete understanding of colistin resistance and its epidemiology. Our molecular study might have provided more accurate data on the true prevalence of the *mcr*. However, the data did not enable us to determine the bacterial species that carried the resistance genes, or the location of the *mcr* in plasmids or in the bacterial chromosomes.

The *mcr-1* gene has spread to most continents, and has been detected in various bacterial isolates from animals, human and the environment, including *Escherichia coli*, *Klebsiella pneumoniae*, *Enterobacter cloacae* and *Enterobacter aerogenes* [30]. The *mcr-2* gene was found on rarely occasion, in *Escherichia*. *coli* isolates from porcine of Belgium and in flies of China [4, 22]. After its first characterization on a IncHI2-type plasmid, pWJ1, from *Escherichia coli* isolated from a Chinese pig [5], the *mcr*-3 gene was shown to be present in bacteria isolated from humans in Denmark, chickens and flies in China, pigs in Japan and cattle in Spain [20–22, 26, 31].

As far as we know, the *mcr-4* gene was first detected in two *Salmonella enterica* serovar Typhimurium strains isolated from human fecal samples and in *Salmonella* and *E*. *coli* isolated from pigs in Italy, Spain and Belgium [25]. After the initially discovery of the *mcr-5* gene in *Salmonella enterica* subsp. *enterica* serovar Paratyphi B, the gene was detected in *Escherichia coli* from diseased pigs and healthy pigs in Japan [26].

Our findings of very high prevalences of the *mcr-4* and *mcr-5* in pigs and poultry from large areas in China are most likely associated with the prolonged and widespread use of colistin as a growth promoter in food animals in China. However, it should be noted that these two genes might also be more prevalent in other countries as few studies looked for them.

It is noticeable that the prevalences of the *mcr-4* and *mcr-5* were generally significantly higher in the nasal/oropharyngeal swabs than in the anal/cloacal swabs in both pigs and poultry. This suggests that bacteria in saliva and respiratory secretions might play important roles in the maintenance and transmission of colistin resistance genes in pigs and poultry. Further comparative studies are needed to determine the bacterial species carrying the *mcr-4* and *mcr-5* in the upper and lower alimentary tract and how there might be transmission of the resistance genes between these populations.

Compared to the reported sequences (MF543359; MG581979) [23, 25], the *mcr-4* identified in this study demonstrated nucleotide mutations (MF543359: 2/1,165; MG581979: 3/1,165), resulting in change in amino acids (MF543359: 2/338; MG581979: 3/338). The alignment of *mcr-5* genes in this study with the initial sequence from *Salmonella* species in German and *E*. *coli* in Japan [24, 26] demonstrated that they were identical.

In conclusions, our study further confirms the presence of the *mcr-4* and *mcr-5* in bacteria from animals and indicates that these genes are widespread in food producing animals (pigs and poultry) in China. Future studies are needed to characterize the bacteria carrying the *mcr-4* and *mcr-5* and their locations on plasmids and/or the bacterial chromosomes.

## Supporting information

S1 Table List of farms from where swine swabs were collected for *mcr* detection in this study. (DOCX)

S2 Table Prevalences of *mcr* in anal (A) and nasal (N) swabs in pigs. (DOCX)

S3 Table Prevalences of *mcr* in cloacal (C) and oropharyngeal (O) swabs in chickens. (DOCX)

S4 Table Prevalences of *mcr* in cloacal (C) and oropharyngeal (O) swabs in ducks. (DOCX)

S5 Table Prevalences of *mcr* in cloacal (C) and oropharyngeal (O) swabs in geese. (DOCX)

S6 Table Prevalences of *mcr* in cloacal (C) and oropharyngeal (O) swabs of pigeons. (DOCX)
