# Supplementary material for: Newly identified colistin resistance genes, mcr-4 and mcr-5, from upper and lower alimentary tract of pigs and poultry in China
Source: PLoS One. 2018 Mar 14;13(3):e0193957. doi: 10.1371/journal.pone.0193957 (PMC5851611; doi:10.1371/journal.pone.0193957)
Supplement: S1 Table — (DOCX) [file pone.0193957.s001.docx]

**S1 Table. List of farms from where swine swabs were collected for *mcr* detection in this study.**

| **Province** | **City** | **Animal #** | **Sample Type** | **Farm** | **Location** |
| --- | --- | --- | --- | --- | --- |
| Guangdong | Jiangmen | 40 | Nasal swab | Private enterprise | N22°34′, E113°04′ |
| Heilongjiang | Haerbin | 60 | Nasal swab | Private enterprise | N45°48′, E126°31′ |
| Henan | Xihua | 63 | Nasal swab | Private enterprise | N33°46′, E114°31′ |
| Jiangsu | Yangzhou | 51 | Anal swab | Abattoir of Yangzhou | N32°23′, E119°24′ |
|  | Dafeng | 37 | Nasal swab | Dafeng Hongguang Livestock and Poultry Cooperative, Jiangsu | N32°58′, E120°48′ |
|  | Jiangyan | 31 | Nasal swab | Jiangyan Wanwei Breeding Co., Ltd., Jiangsu | N32°30′, E120°07′ |
|  | Liyang | 23 | Nasal swab | Liyang Daitou Wanfeng Livestock and Pourty Breeding Co., Ltd., Jiamgsu | N31°25′, E119°28′ |
|  | Nanjing | 394 | Nasal swab | Nanjing Zhoubang Biotechnology Co., Ltd., Jiangsu | N32°03′, E118°47′ |
|  | Taixing | 18 | Nasal swab | Taixing Hongtai Pig Farm, Jiangsu | N32°10′, E120°02′ |
|  | Wuxi | 36 | Nasal swab | Wuxi Hongsheng Livestock and Poultry Breeding Co., Ltd., Jiangsu | N31°29′, E120°18′ |
| Jilin | Changchun | 63 | Nasal swab | Private enterprise | N43°48′, E125°19′ |
| Shandong | Jining | 60 | Nasal swab | Private enterprise | N35°24′, E116°34′ |
| Shanghai | Shanghai | 53 | Nasal swab | Shanhai Livestock Farm | N31°13′, E121°28′ |
| Yunnan | Kunming | 66 | Anal/Nasal swab | Private enterprise | N24°52′, E102°49′ |
| Zhejiang | Ningbo | 98 | Anal/Nasal swab | Private enterprise | N29°52′, E121°32′ |
|  | Shaoxing | 345 | Anal/Nasal swab | Private enterprise | N30°01′, E120°34′ |
