# Supplementary material for: Newly identified colistin resistance genes, mcr-4 and mcr-5, from upper and lower alimentary tract of pigs and poultry in China
Source: PLoS One. 2018 Mar 14;13(3):e0193957. doi: 10.1371/journal.pone.0193957 (PMC5851611; doi:10.1371/journal.pone.0193957)
Supplement: S2 Table — (DOCX) [file pone.0193957.s002.docx]

**S2 Table. Prevalences of *mcr* in anal (A) and nasal (N) swabs in pigs.**

| **Province** | **City** | **Positive /total samples** | |
| --- | --- | --- | --- |
|  |  | ***mcr-4*** | ***mcr-5*** |
| Jiangsu | Yangzhou | A: 2/51 | A: 6/51 |
| Yunnan | Kunming | A: 6/64  N: 35/66  *T: 41/130 | A: 9/64  N: 18/66  T: 27/130 |
| Zhejiang | Ningbo | A: 26/48  N: 40/50  T: 47/50 | A: 42/48  N: 43/50  T: 50/50 |
|  | Shaoxing | A: 3/50  N: 149/345  T:150/345 | A: 2/50  N: 252/345  T:253/345 |
| Guangdong | Jiangmen | N: 8/40 | N: 11/40 |
| Heilongjiang | Haerbin | N: 15/60 | N: 4/60 |
| Henan | Xihua | N: 35/63 | N: 8/63 |
| Jiangsu | Dafeng | N: 32/37 | N: 13/37 |
|  | Jiangyan | N: 11/31 | N: 1/31 |
|  | Liyang | N: 19/23 | N: 9/23 |
|  | Nanjing | N: 141/394 | N: 54/394 |
|  | Taixing | N: 12/18 | N: 17/18 |
|  | Wuxi | N: 21/36 | N: 9/36 |
| Jilin | Changchun | N: 33/63 | N: 2/63 |
| Shandong | Jining | N: 3/60 | N: 8/60 |
| Shanghai | Shanghai | N: 51/53 | N: 6/53 |

*T: total number of assayed animals.
