# Supplementary material for: Newly identified colistin resistance genes, mcr-4 and mcr-5, from upper and lower alimentary tract of pigs and poultry in China
Source: PLoS One. 2018 Mar 14;13(3):e0193957. doi: 10.1371/journal.pone.0193957 (PMC5851611; doi:10.1371/journal.pone.0193957)
Supplement: S3 Table — (DOCX) [file pone.0193957.s003.docx]

**S3 Table. Prevalences of *mcr* in cloacal (C) and oropharyngeal (O) swabs in chickens.**

| **Province** | **City** | **Positive /total samples** | |
| --- | --- | --- | --- |
|  |  | ***mcr-4*** | ***mcr-5*** |
| Anhui | Fuyang | C: 2/34;  O: 3/34;  T: 4/34 | *T: 0/34 |
| Fujian | Nanping | C: 3/35;  O: 3/35;  T: 6/35 | C: 1/35;  O: 0/35;  T: 1/35 |
| Gansu | Jingyuan | C:2/57;  O: 0/57;  T: 2/57 | T: 0/57 |
| Guangdong | Zhanjiang | C: 21/65;  O: 3/54;  T: 24/65 | C: 5/65;  O: 5/54;  T: 10/65 |
| Guangxi | Beihai | C: 12/65;  O: 11/65;  T: 23/130 | C: 5/65;  O: 7/65;  T: 12/130 |
| Hainan | Wenchang | C: 3/70;  O: 3/70;  T: 6/70 | C: 0/70;  O: 15/70;  T: 15/70 |
| Hebei | Shijiazhuang | C: 21/46;  O: 5/50;  T: 26/96 | C: 2/46;  O: 1/50,  T: 3/96 |
| Henan | Anyang | C: 5/56;  O: 7/56;  T: 11/56 | C: 10/56;  O: 2/56;  T: 12/56 |
| Hubei | Wuhan | C: 9/64;  O: 6/53;  T: 13/64 | C: 1/64;  O: 2/53;  T: 3/64 |
| Hunan | Yongzhou | T: 0/70 | C: 0/70;  O: 8/70;  T: 8/70 |
| Inner Mongolia | Ulanqab | C: 0/65;  O: 9/65;  T: 9/65 | C: 0/65;  O: 6/65;  T: 6/65 |
| Jiangsu | Yangzhou | C: 2/126;  O: 4/111;  T: 6/126 | C: 14/126;  O: 6/111;  T: 19/126 |
|  | Yixing | C: 1/28;  O: 4/28;  T: 5/28 | C: 0/28;  O: 1/28;  T: 1/28 |
| Jiangxi | Xingan | C: 4/49;  O: 17/49;  T: 18/49 | C: 5/49;  O: 21/49;  T: 24/49 |
| Jilin | Changchun | C: 16/70;  O: 9/70;  T: 24/70 | C: 4/70;  O: 4/70;  T: 8/70 |
| Liaoning | Jinzhou | C: 1/37;  O: 9/37;  T: 10/37 | C: 1/37;  O: 2/37;  T: 3/37 |
| Shaanxi | Yanan | T: 0/70 | T: 0/70 |
| Shandong | Liaocheng | C: 0/59;  O: 1/59;  T: 1/59 | T: 0/59 |
| Shanxi | Changzhi | C: 18/20;  O: 17/20;  T: 20/20 | C: 1/20;  O: 3/20;  T: 4/20 |
| Sichuan | Chengdu | C: 7/70;  O: 2/70;  T: 9/70 | C: 3/70;  O: 0/70;  T: 3/70 |
| Tibet | Shigatse | C: 2/30;  O: 3/30;  T: 5/30 | T: 0/30 |
| Xinjiang | Hoboksar | C: 6/70;  O: 11/70;  T: 17/70 | C: 1/70;  O: 3/70;  T: 4/70 |
| Yunnan | Yiwei | C: 1/70;  O: 9/70;  T: 10/70 | C: 0/70;  O: 7/70;  T: 7/70 |
| Zhejiang | Wenzhou | C: 7/57;  O: 1/57;  T: 8/57 | C: 5/57;  O: 0/57;  T: 5/57 |

*T: total number of assayed animals.
