# Supplementary material for: Newly identified colistin resistance genes, mcr-4 and mcr-5, from upper and lower alimentary tract of pigs and poultry in China
Source: PLoS One. 2018 Mar 14;13(3):e0193957. doi: 10.1371/journal.pone.0193957 (PMC5851611; doi:10.1371/journal.pone.0193957)
Supplement: S4 Table — (DOCX) [file pone.0193957.s004.docx]

**S4 Table. Prevalences of *mcr* in cloacal (C) and oropharyngeal (O) swabs in ducks.**

| **Province** | **City** | **Positive /total samples** | |
| --- | --- | --- | --- |
|  |  | ***mcr-4*** | ***mcr-5*** |
| Fujian | Nanping | C: 2/33;  O: 0/33;  *T: 2/33 | C: 1/33;  O: 0/33;  T: 1/33 |
| Guangdong | Zhanjiang | T: 0/4 | T: 0/4 |
| Guangxi | Beihai | C: 0/5;  O: 3/5;  T: 3/10 | C: 0/5;  O: 1/5;  T: 1/10 |
| Hebei | Shijiazhuang | C: 1/3;  O: 1/3;  T: 2/6 | T: 0/6 |
| Henan | Anyang | C: 1/7;  O: 0/7;  T: 1/7 | T: 0/7 |
| Hubei | Wuhan | C: 1/6  O: 3/6  T: 4/6 | T: 0/6 |
| Jiangsu | Yangzhou | C: 1/10;  O: 0/10;  T: 1/10 | C: 0/10;  O: 2/10;  T: 2/10 |
|  | Yixing | C: 1/21  O: 1/21  T: 2/21 | C: 2/21  O: 2/21  T: 3/21 |
| Jiangxi | Xingan | C: 0/11;  O: 1/11;  T: 1/11 | C: 0/11;  O: 3/11;  T: 3/11 |
| Liaoning | Jinzhou | T: 0/7 | T: 0/7 |
| Shandong | Liaocheng | C: 1/3;  O: 0/3;  T: 1/3 | T: 0/3 |
| Zhejiang | Wenzhou | C: 3/12;  O: 0/12;  T: 3/12 | T: 0/12 |

*T: total number of assayed animals.
