# Supplementary material for: Newly identified colistin resistance genes, mcr-4 and mcr-5, from upper and lower alimentary tract of pigs and poultry in China
Source: PLoS One. 2018 Mar 14;13(3):e0193957. doi: 10.1371/journal.pone.0193957 (PMC5851611; doi:10.1371/journal.pone.0193957)
Supplement: S5 Table — (DOCX) [file pone.0193957.s005.docx]

**S5 Table. Prevalences of *mcr* in cloacal (C) and oropharyngeal (O) swabs in geese.**

| **Province** | **City** | **Positive /total samples** | |
| --- | --- | --- | --- |
|  |  | ***mcr-4*** | ***mcr-5*** |
| Henan | Anyang | C: 1/7  O: 2/7  *T: 3/7 | C: 1/7  O: 0/7  T: 1/7 |
| Inner Mongolia | Ulanqab | T: 0/5 | T: 0/5 |
| Jiangsu | Yangzhou | C: 3/9;  O: 2/9;  T: 5/9 | C: 2/9;  O: 7/9;  T: 7/9 |
| Jiangxi | Xingan | C: 1/9;  O: 0/9;  T: 1/9 | C: 1/9;  O: 1/9;  T: 2/9 |
| Shandong | Liaocheng | C: 0/8  O: 1/8  T: 1/8 | T: 0/8 |
| Shanghai | Shanghai | C: 25/70;  O: 32/70;  T: 44/70 | C: 2/70;  O: 8/70;  T: 9/70 |
| Zhejiang | Wenzhou | T: 0/1 | T: 0/1 |

*T: total number of assayed animals.
