# Supplementary material for: Newly identified colistin resistance genes, mcr-4 and mcr-5, from upper and lower alimentary tract of pigs and poultry in China
Source: PLoS One. 2018 Mar 14;13(3):e0193957. doi: 10.1371/journal.pone.0193957 (PMC5851611; doi:10.1371/journal.pone.0193957)
Supplement: S6 Table — (DOCX) [file pone.0193957.s006.docx]

**S6 Table. Prevalences of *mcr* in cloacal (C) and oropharyngeal (O) swabs of pigeons.**

| **Province** | **City** | **Positive /total samples** | |
| --- | --- | --- | --- |
|  |  | ***mcr-4*** | ***mcr-5*** |
| Gansu | Jingyuan | *T: 0/13 | T: 0/13 |
| Hebei | Shijiazhuang | T: 8/17  T: 2/17  T: 10/34 | T: 1/17  T: 0/17  T: 1/34 |
| Jiangsu | Yangzhou | C: 1/25;  O: 2/10;  T: 3/25 | T: 0/25 |
|  | Yixing | C: 1/21  O: 1/20  T: 2/21 | C: 1/21  O: 0/20  T: 1/21 |
| Liaoning | Jinzhou | C: 1/6  O: 1/6  T: 2/6 | C: 0/6  O: 1/6  T: 1/6 |

*T: total number of assayed animals.
